# GV effects of diabetes mellitus on clinical outcomes of patients with acute heart failure: A systematic review and meta-analysis

**DOI:** 10.1371/journal.pone.0338653

**Published:** 2025-12-10

**Authors:** Linna Zhao, Juanjuan Zhang, Weizhe Liu, Cheng Dai, Aiying Li

**Affiliations:** 1 Department of Biochemistry and Molecular Biology, College of Basic Medicine, Hebei University of Chinese Medicine, Shijiazhuang, Hebei, China; 2 Hebei Key Laboratory of Chinese Medicine Research on Cardio-cerebrovascular Disease, Shijiazhuang, Hebei, China; 3 Hebei Higher Education Institute Applied Technology Research Center on TCM Formula Preparation, Shijiazhuang, Hebei, China; Menzies School of Health Research: Charles Darwin University, AUSTRALIA

## Abstract

Diabetes mellitus (DM) is identified as a potential modifier of clinical outcomes in acute heart failure (AHF), yet its prognostic impact is not fully determined. This systematic review and meta-analysis aimed to assess the prognostic impact of DM on survival outcomes in AHF patients by synthesizing evidence from 26 studies involving 326,928 subjects collected from Cochrane Library, PubMed, Web of Science, and Embase databases up to 1 June 2024. Both prospective/retrospective cohort and case-control studies published since 2000 were included, with outcomes evaluated through multivariate, univariate, and binary analyses using the Newcastle-Ottawa Scale for quality assessment. Multivariate analysis indicated that DM significantly increased the risk of all-cause mortality in AHF patients (cohort studies: HR = 1.21, 95%CI (1.13, 1.29), OR=1.15, 95%CI (1.05, 1.26); case-control studies: HR = 1.39, 95%CI (1.26, 1.53), OR=1.43, 95%CI (1.10, 1.84)]. Univariate analysis confirmed this finding in case-control studies [HR = 1.30, 95%CI (1.01, 1.67)], but not in cohort studies. In both cohort [RR = 1.27, 95%CI (1.12, 1.43)] and case-control [OR=1.21, 95%CI (1.08, 1.35)] studies, DM increased the risk of all-cause mortality. AHF patients with DM had a higher risk of cardiovascular mortality [cohort studies: HR = 1.85, 95%CI (1.46, 2.33); case-control: OR=1.70, 95%CI (1.17, 2.47)]. While multivariate analysis showed no association between DM and in-hospital mortality, case-control studies indicated an increased risk [OR=1.21, 95%CI (1.03, 1.42)]. DM also increased the risk of readmission [cohort studies: HR = 1.32, 95%CI (1.14, 1.53); case-control studies: HR = 1.44, 95%CI (1.23, 1.69); binary data: OR=1.19, 95%CI (1.07, 1.31)].This updated meta-analysis demonstrates that DM imposes significant adverse effects on all-cause mortality, cardiovascular-related mortality, and readmission risk in AHF patients. However, no significant connection was found between diabetes and survival outcomes with respect to the co-endpoint of death or readmission and the endpoint of in-hospital mortality. These findings underscore the necessity for implementing targeted diabetes management within AHF care protocols to enhance clinical outcomes, an essential consideration for future practice.

## 1 Introduction

Acute heart failure (AHF) is a sudden clinical syndrome resulting from various factors. The symptoms and signs of heart failure rapidly surface or worsen abruptly, along with increased plasma levels of natriuretic peptides, often posing a life-threatening risk [1–3]. Despite advancements in management, the prognosis for AHF continues to be unfavorable, with a 12% mortality rate during hospital stay, approximately a 45% readmission rate within one year, and a 22% mortality rate within the same period [4]. Therefore, AHF is a significant global public health issue, severely impacting patients’ quality of life and posing a huge burden on healthcare systems.

Diabetes mellitus (DM) is a long-term metabolic disorder. It is important to mention that diabetes has a strong connection to cardiovascular diseases, particularly in patients with AHF, where its prevalence is notably higher than in the general population. The prevalence of diabetes in AHF patients is a significant concern in medical practice. As reported by the ESC Heart Failure Long-term Registry, the prevalence of diabetes among ADHF inpatients is 39.1%, with rates ranging from 33.8% in patients with cardiogenic shock to 43.2% in those with AHF and acute coronary syndrome (ACS) [5]. DM influences AHF through a variety of interconnected mechanisms that result in hemodynamic instability, metabolic dysregulation, and acute inflammatory-ischemic episodes. Hyperglycemia, insulin resistance, and diabetic comorbidities collectively destabilize cardiac function through direct myocardial injury, impaired cellular energetics, and neurohormonal activation. Acute hyperglycemia causes osmotic diuresis and intravascular volume depletion, prompting compensatory neurohormonal responses that increase myocardial oxygen demand, decrease coronary perfusion, and trigger ischemia in vulnerable patients, especially those with concurrent ACS [6,7]. This ischemia-reperfusion injury, alongside DM-related endothelial dysfunction and reduced nitric oxide availability, worsens myocardial stunning and diastolic dysfunction, leading to acute pulmonary congestion [8]. DM-induced metabolic disturbances further impair cardiac contractility. Hyperglycemia rapidly increases free fatty acid (FFA) flux, suppressing glucose use and forcing cardiomyocytes to depend on inefficient FFA oxidation, which drains adenosine triphosphate (ATP) stores and worsens intracellular calcium mismanagement—a key factor in systolic dysfunction [9]. Concurrent insulin resistance diminishes insulin’s anti-apoptotic and vasodilatory effects, fostering cardiomyocyte apoptosis and microvascular rarefaction. These processes are intensified by acute hyperglycemia-induced oxidative stress, which produces reactive oxygen species (ROS) that directly damage mitochondrial membranes, disrupt sarcomeric proteins, and activate matrix metalloproteinases (MMPs), destabilizing extracellular matrix structure and promoting myocardial edema [10,11]. Inflammatory cascades play a central role in acute decompensation. DM establishes a proinflammatory state marked by increased interleukin-6 (IL-6), tumor necrosis factor-alpha (TNF-α), and C-reactive protein (CRP), which acutely enhance endothelial permeability, promote leukocyte infiltration, and induce cardiomyocyte cytokine toxicity [12,13]. This systemic inflammation intensifies volume overload by increasing capillary leak and lowering oncotic pressure, especially in patients with hypoalbuminemia, a frequent characteristic of diabetic AHF [12]. Additionally, acute hyperglycemia impairs sodium-potassium ATPase activity, leading to intracellular sodium and calcium overload, which predisposes individuals to arrhythmias and further compromises myocardial contractility [14,15].

Due to diabetes potentially leading to serious adverse outcomes, its impact on increasing the risk of adverse prognosis in AHF patients has garnered significant attention from researchers. Given the complex pathophysiological interaction between diabetes and AHF, investigating effective therapeutic strategies has become a critical clinical necessity. Studies have demonstrated the positive effects of certain antidiabetic medications on the prognosis of heart failure patients. Traditional mineralocorticoid receptor antagonists (MRAs) like spironolactone and eplerenone effectively lower cardiovascular mortality in chronic heart failure but carry hyperkalemia risks in AHF with diabetes, particularly in those with renal impairment. The use of insulin during hospitalization might exacerbate cardiorenal outcomes through electrolyte imbalances. Nonsteroidal MRAs provide similar benefits with a reduced risk of hyperkalemia, potentially offering advantages in managing AHF with diabetes. Spironolactone can temporarily elevate HbA1c levels, while eplerenone exhibits a neutral metabolic impact. [16] Consequently, further research is required to define the optimal selection and timing of MRAs in the AHF-diabetes cohort, particularly concerning interactions with emerging therapies such as SGLT2 inhibitors. In a previous meta-analysis, researchers also attempted to review the association between diabetes and AHF [17]. However, in their research, the primary focus is on CHF, with very limited evidence addressing AHF. Additionally, only the association between AHF and all-cause mortality was reviewed, with a paucity of systematic evidence for other adverse outcomes, posing certain challenges for preventing adverse prognosis in AHF. Hence, this study was conducted to examine the impact of diabetes on the clinical outcomes of AHF patients, aiming to provide a robust evidence-based foundation for improving patient prognosis.

## 2 Materials and methods

### 2.1 Inclusion and exclusion criteria

This meta-analysis followed the Cochrane Handbook for Systematic Reviews of Interventions (for details, see http://training.cochrane.org/handbook) and the Preferred Reporting Items for Systematic Reviews and Meta-Analyses (File S1) [18]. This study protocol was registered with PROSPERO (CRD: CRD42023420341).

#### 2.1.1 *Study type.*

Cohort studies and case-control studies.

#### 2.1.2 *Subjects.*

(1) Age > 18 years; (2) Patients diagnosed with AHF.

#### 2.1.3 *Exposure factors.*

1. AHF combined with DM of the exposure group; 2. AHF alone of the control group.

#### 2.1.4 *Outcome indicators.*

The primary outcome indicators included all-cause mortality, in-hospital mortality, cardiovascular mortality, and readmission; while the secondary outcome indicators were death and readmission.

#### 2.1.5 *Exclusion criteria.*

1. Diseases outside the scope of this study to be explored 2. Study data are unavailable for acquisition 3. Reviews, pathological studies, animal experiments, dissertations, or conference papers 4. Patients with other significant diseases, such as recent myocardial infarction, unstable angina pectoris, and end-stage renal disease

### 2.2 Search strategy

We conducted a search in the Cochrane Library, PubMed, Web of Science, and Embase databases from their inception until June 1, 2024, for literature on the prognostic factors of patients with DM combined with AHF. The search utilized both subject terms and free terms, including “ DM “ and “AHF” as search terms. There were no language or regional limitations imposed. S1 Table provides a detailed explanation of the search strategy.

### 2.3 Screening of literature and extraction of data

Based on the inclusion and exclusion criteria, two investigators reviewed the literature and imported the relevant documents into Endnote 20 to remove duplicates. They reviewed the titles or abstracts to identify potentially eligible studies and downloaded the entire texts. After reading the complete texts, original studies suitable for this systematic review were included. The two investigators independently extracted the data, cross-verifying them with consistent units, and referred disputed articles to a third researcher for consensus resolution. Data extraction was conducted on June 5, 2024. The extracted information primarily consisted of the title, first author, publication year, country of residence, study type, exposure factor, sample size, gender of exposure and control groups, patient age, follow-up time, and outcome indicators. When essential information was missing, we contacted the original study authors to obtain the supplementary data.

### 2.4 Risk of bias assessment on the included studies

Two investigators independently evaluated the risk of bias in the included studies using the Newcastle Ottawa Scale (NOS), subsequently cross-checking the results. In cases of disagreement, decisions were reached via discussion or with the intervention of a third party. For case-control studies, the assessment criteria included the appropriateness of the case’s definition and diagnosis, the representativeness of the case, the definition and comparability of the control group, the investigation and assessment methods of the exposure, the uniformity in investigation methods between the case and control groups, and the non-response rate. For cohort studies, the evaluation criteria comprised the representativeness of the exposure cohort, the selection of the non-exposure cohort, the determination of exposure, ensuring no subjects had developed the disease under study at the study’s inception, comparability, the determination method of results, the sufficiency of follow-up time for the disease under study, and the adequacy of follow-up. According to the NOS-defined assessment, studies receiving more than 7 stars were classified as high-quality, while those receiving 5–7 stars and 0–4 stars were classified as medium-quality and low-quality, respectively.

Two researchers independently utilized NOS for quality evaluation. Upon completion, cross-checks were performed. In case of disagreements, a third researcher was called upon to assist in the decision-making process.

### 2.5 Assessment of the quality of the evidence

Our evaluation of the evidence quality in the included studies employed the Grading of Recommendations Assessment, Development and Evaluation (GRADE) system [19]. Widely adopted, GRADE is a tool for assessing the quality of evidence concerning outcome measures in systematic reviews, categorizing evidence into four levels: high, moderate, low, and very low. This system conducts a thorough evaluation of factors like risk of bias, inconsistency, indirectness, imprecision, and publication bias to determine the credibility of effect estimates for specific interventions.

### 2.6 Method of statistics

The statistical analysis was performed using Stata 15.0, encompassing heterogeneity tests, publication bias analyses, and sensitivity analyses. The effect values, HR (Hazard Ratio) and OR (Odds Ratio), from the included studies were combined separately, and the 95% confidence interval (CI) was calculated. Binary data were pooled using the OR (Odds Ratio) with a 95% CI. The Q statistic and *I²* test were utilized to evaluate heterogeneity. *P* > 0.1 and *I²* ≤ 50% suggest acceptable heterogeneity across studies, so a fixed-effects model was applied for the meta-analysis; P ≤ 0.1 or *I²* > 50% indicates substantial statistical heterogeneity across studies, thereby a random effects model was used for the meta-analysis. The “metabias” command was employed to assess the publication bias in the included studies. Results with *P* < 0.05 were deemed statistically significant. The analysis data of each outcome indicator are provided in S2 Table.

## 3 Results

### 3.1 Literature screening

**Fig 1:**
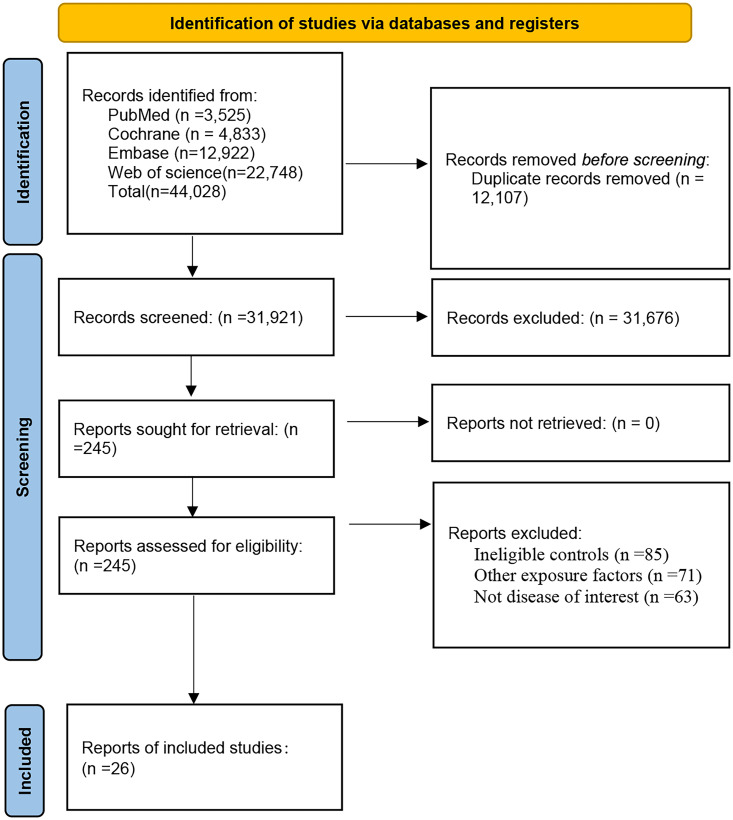
The literature screening process and results.

A total of 38,626 articles were retrieved. Of these, 11,170 were excluded due to duplication, 27,211 were dismissed after reviewing titles and abstracts as irrelevant, and 219 were disregarded after reading the full texts (refer to S3 Table). Ultimately, 26 studies were included. Fig 1 illustrates the literature screening process and outcomes.

### 3.2 Basic characteristics of included articles

In total, 26 studies were encompassed, including 11 case-control studies and 15 cohort studies, and involving 326,928 participants, with 112,506 individuals in the exposed group and 214,422 individuals in the control group. One study originated from California, 3 were from China, 1 from the Czech Republic, 1 from Finland, 2 from Italy, 4 from Japan, 5 from Multicenter, 1 from Saudi Arabia, 1 from Singapore, 1 from Spain, and 4 from the USA. Nineteen studies assessed the all-cause mortality risk, 2 investigated cardiovascular mortality risk, 5 researched in-hospital mortality risk, 6 studied readmission risk, and 5 examined the composite endpoint risk of mortality or readmission. The average age range was from 53 to 85. None of the studies addressed whether antidiabetic drugs would reduce the risk of AHF occurrence in diabetic patients.

All the relevant articles were written in English. The basic characteristics of the included articles are provided in Table 1.

**Table 1:** Basic characteristics of included studies.

Study (name, year)	Country	Quality assessment (NOS)		Study type	Exposure factor	Sample size(E/C)	Age (E/C)	Disease	Outcomes
		Case Control	Cohort						
Juana A Flores-Le Roux,2011	Spain	6		Case Control Study	Diabetes	149/188	71.4 (7.7)/ 71.1 (11.4)	AHF	①②
John T. Parissis,2012	Multicenter	5		Case Control Study	Diabetes	2229/ 2724	–	AHF	③
Zachary L. Cox,2020	USA	6		Case Control Study	Diabetes	736/ 309	75 (7.0)/ 77 (7.5)	AHF	①
Yun Yun Go, 2014	Singapore	7		Case Control Study	Diabetes	1108/ 1013	69.1(11.1)/70.0 (13.3)	AHF	① ③④
Sylvestre Mare´chaux Marie M, 2011	France		4	Cohort Study	Diabetes	47/51	76(9)	AHF	⑤
Siu-Hin Wan, 2022	USA	7		Case Control Study	Diabetes	381/ 531	77.3(11.8)/ 80.7(13.8)	AHF	①④
Yu‑Yang Chen, 2018	China	5		Case Control Study	Diabetes	173/347	65.2(10.4)/65.1(13.6)	AHF	①②④
Giovanni Targher, 2017	Multicenter		8	Cohort Study	Diabetes	3422/3504	70.0(11.4)/68.0(14.4)	AHF	①③④
Andrea Fabbri, 2016	Italy		7	Cohort Study	Diabetes	325/909	84.0	AHF	①
Alexandre Mebazaa, 2013	Multicenter	7		Case Control Study	Diabetes	2543/3669	74.1 (3.95)	AHF	①
Masahiro Seo, 2019	Japan		8	Cohort Study	Diabetes	156/215	74(13)	ADHF	①
Akihiro Shirakabe,2018	Japan	5		Case Control Study	Diabetes	538/696	72 (3.5)/ 76 (4.0)	AHF	①
Hideyuki Takimura,2018	Japan	5		Case Control Study	Diabetes	357/834	81.1(11.1)/ 77.9(12.6)	ADHF	④
Marvin Louis Roy Lu, 2016	USA	6		Case Control Study	Diabetes	337/274	68(13)/ 65(16)	ADHF	①④
Damien Logeart, 2008	France		7	Cohort Study	Diabetes	95/321	69(14)/74(11)	AHF	⑤
Kalyani Anil Boralkar,2020	California	6		Case Control Study	Diabetes	157/ 286	76.7(15.5)	AHF	①
Veli-Pekka Harjola,2010	Finland		8	Cohort Study	Diabetes	985/1992	71.7 (4.05)	AHF	①
Rami Khayat, 2015	USA		8	Cohort Study	Diabetes	456/ 661	60.3 (14.7)	AHF	①
Ming-Feng Lee,2012	Taiwan, China		8	Cohort Study	Diabetes	50/81	58 (14)/61(13)	AHF	⑤
Amar Salam,2016	Multicenter		6	Cohort Study	Diabetes	2492/2463	63.5(11.9)/53(16.6)	AHF	①③
Khalid F. AlHabib,2014	Saudi Arabia		8	Cohort Study	Diabetes	1669/ 941	61.4 (15.0)	ADHF	①
Marián Felšöci, 2011	Czech Republic		7	Cohort Study	Diabetes	983/1438	71.6(12.5)	AHF	①
Hadi A. R. Khafaji,2015	Multicenter		7	Cohort Study	Diabetes	2492/ 2513	59(15)/65(12)	AHF	①③
Yang Li, 2021	China		8	Cohort Study	Diabetes	1003/2332	71 (5.25)	AHF	①
Marco Metra, 2012	Italy		7	Cohort Study	Diabetes	79/ 119	68 (12)	AHF	⑤
Takahiro Sakai, 2022	Japan		5	cohort study	Diabetes	110/ 269	85 (2.5)/ 83 (3)	AHF	⑤

Notes: ①All-cause death, ②Cardio-vascular death, ③In-hospital mortality, ④Rehospitalization, ⑤Death or hospitalization. ADHF: Acute decompensate heart failure; AHF: Acute heart failure; E/C: Exposure/Control.

### 3.3 Quality assessment

The quality of the included case-control studies and cohort studies was assessed by NOS. There were 7 high-quality studies [20–26], 18 medium-quality studies [27–44], and 1 low-quality study [45]. The detailed NOS scores of the studies are presented in Table 2.

**Table 2:** NOS scores for case control studies (a) and cohort studies (b).

Study	Year	Selection of cases and controls				V5	Exposure			Total score
		V1	V2	V3	V4		V6	V7	V8	
Juana A Flores-Le Roux	2011	1	1	0	0	1	1	1	1	6
John T. Parissis	2012	1	1	0	0	1	1	1	0	5
Zachary L. Cox	2020	1	1	0	0	1	1	1	1	6
Yun Yun Go	2014	1	1	1	0	1	1	1	1	7
Siu-Hin Wan	2022	1	1	1	0	1	1	1	1	7
Yu-Yang Chen	2018	1	1	0	0	1	1	1	0	5
Alexandre Mebazaa	2013	1	1	1	0	1	1	1	1	7
Akihiro Shirakabe	2018	1	1	0	0	1	1	1	0	5
Hideyuki Takimura	2018	1	1	0	0	1	0	1	1	5
Marvin Louis Roy Lu	2016	1	1	1	0	1	1	1	0	6
Kalyani Anil Boralkar	2020	1	1	1	0	1	1	1	0	6
**Study**	**Year**	**Selection of cohorts**				**V5**	**Outcome**			**Total score**
		**V1**	**V2**	**V3**	**V4**		**V6**	**V7**	**V8**	
Sylvestre Mare´chaux • Marie M	2011	1	0	1	1	0	1	0	0	4
Giovanni Targher	2017	1	1	1	1	1	1	1	1	8
Andrea Fabbri	2016	1	1	1	1	1	1	1	0	7
Masahiro Seo	2019	1	1	1	1	1	1	1	1	8
Damien Logeart	2008	1	1	1	1	1	1	1	0	7
Veli-Pekka Harjola	2010	1	1	1	1	1	1	1	1	8
Rami Khayat	2015	1	1	1	1	1	1	1	1	8
Ming-Feng Lee	2012	1	1	1	1	1	1	1	1	8
Amar Salam	2016	1	1	1	1	0	1	1	0	6
Khalid F. AlHabib	2014	1	1	1	1	1	1	1	1	8
Marián Felšöci	2011	1	1	1	1	0	1	1	1	7
Hadi A. R. Khafaji	2015	1	1	1	1	1	1	1	0	7
Yang Li	2021	1	1	1	1	1	1	1	1	8
Marco Metra	2012	1	1	1	1	1	1	1	0	7
Takahiro Sakai	2022	1	1	0	1	0	1	0	1	5

Notes: V1-Is the case definition adequate, V2-Representativeness of the cases, V3-Selection of controls, V4-Definition of controls, V5-Comparability, V6-Ascertainment and assessment of exposure, V7-Are the assessment methods were the same for cases and controls, V8-Non-response rate.

Notes: V1-Representativeness of exposed cohort, V2-Selection of non-exposed cohort, V3-Ascertainment of exposure, V4-Outcome of interest was not present at start of study, V5-Comparability, V6-Assessment of outcome, V7-Was follow-up long enough for outcomes to occur, V8-Adequacy of follow up.

### 3.4 Results of meta-analysis

#### 3.4.1 Risk of death.

(1) *Results of multivariate meta-analysis of all-cause mortality*

**Fig 2:**
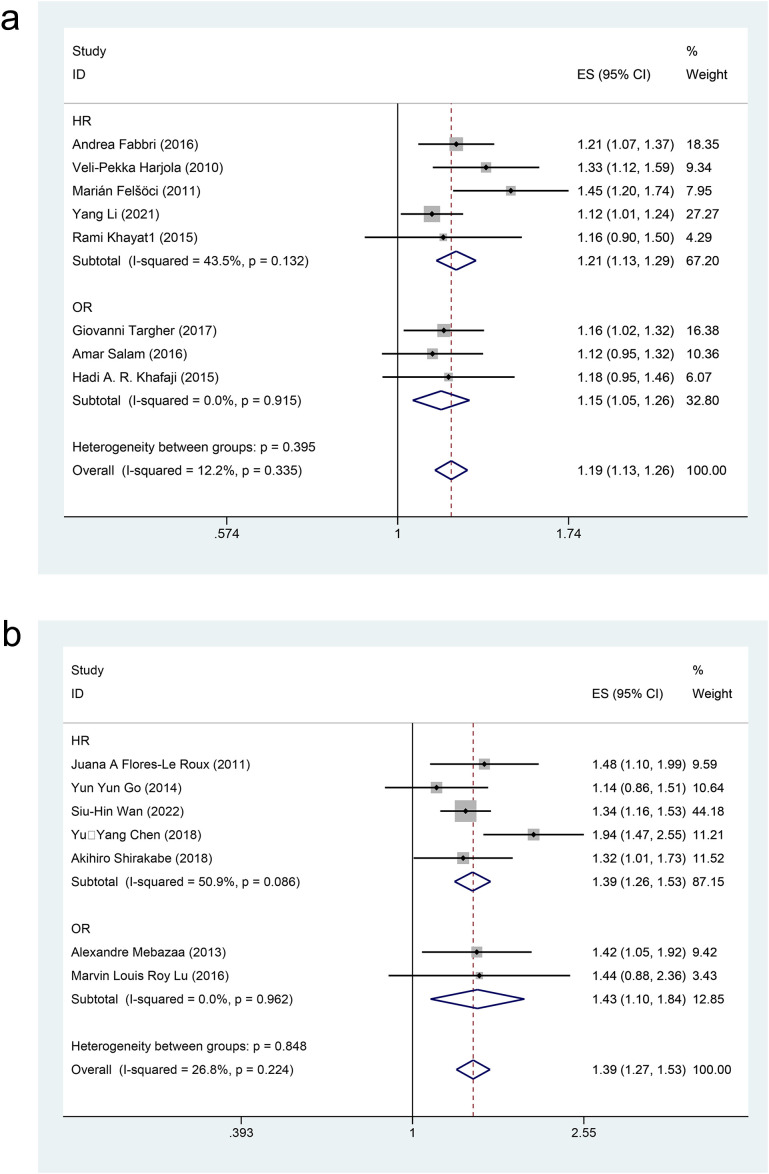
Forest map of multivariate meta-analysis of all-cause mortality (a) in the cohort studies and (b) in the case-control studies.

By utilizing multivariate analysis, eight cohort studies [20,22,23,26,27,29–31] and seven case-control studies [34,37–41,43] evaluated the impact of DM on all-cause mortality among patients with AHF, involving 17,827 patients in the exposure group compared to 22,090 in the control group. The effect sizes of cohort studies (*I²* = 12.2%, *P* = 0.335) and case-control studies (*I²* = 26.8%, *P* = 0.224) were pooled separately using a fixed-effects model. Subgroup analyses were conducted according to study type. Results indicated that in cohort studies [HR = 1.21, 95%CI (1.13, 1.29), GRADE = Moderate] [OR=1.15, 95%CI (1.05, 1.26), GRADE = Moderate] and in case-control studies [HR = 1.39, 95%CI (1.26, 1.53), GRADE = Moderate] [OR=1.43, 95%CI (1.10, 1.84), GRADE = Moderate], DM significantly increased the risk of all-cause mortality in patients with AHF, as illustrated in Fig 2 (a) Cohort study, (b) case-control study and Table 3.

**Table 3:** Meta-analysis results of each outcome measure and GRADE ratings.

Outcomes	Design	Studies	Effect size(95%CI)	I2(%)	P(I2)	GRADE score
multivariate OS	cohort-HR	5	1.21(1.13-1.29)	43.5	0.132	⊕⊕⊕⊙Moderate
	cohort-OR	3	1.15(1.05-1.26)	0	0.915	⊕⊕⊕⊙Moderate
	case control-HR	5	1.39(1.26-1.53)	50.9	0.086	⊕⊕⊕⊙Moderate
	case control-OR	2	1.43(1.10-1.84)	0	0.962	⊕⊕⊕⊙Moderate
univariate OS	cohort	3	1.13(0.94-1.36)	44.5	0.165	⊕⊕⊕⊙Moderate
	case control	2	1.61(0.99-2.60)	77.4	0.036	⊕⊕⊙⊙Low
Cardiovascular Mortality		2	1.85(1.46-2.33)	0	0.548	⊕⊕⊕⊙Moderate
In-Hospital Mortality						
	cohort	2	1.36(0.82-2.26)	81.4	0.02	⊕⊕⊙⊙Low
	case control	1	1.18(0.86-1.62)	NA	NA	⊕⊕⊙⊙Low
Readmission Rate	cohort	3	1.44(1.23-1.69)	32.8	0.226	⊕⊕⊕⊙Moderate
	case control	1	1.32(1.14-1.53)	NA	NA	⊕⊕⊙⊙Low
Death or Readmission		5	1.35(0.94-1.94)	67.7	0.015	⊕⊕⊙⊙Low
Binary Mortality Data of In-hospital mortality	cohort	2	1.27(1.12-1.43)	53.4	0.143	⊕⊕⊕⊙Moderate
	case-control	3	1.21(1.03-1.42)	0	0.513	⊕⊕⊕⊙Moderate
Binary Mortality Data of All-cause mortality	case-control	4	1.21(1.08-1.35)	67.4	0.027	⊕⊕⊙⊙Low
Binary Mortality Data of All-Cardiovascular mortality	case-control	1	1.70(1.17-2.47)	NA	NA	⊕⊕⊙⊙Low
Binary Readmission Rate Data		3	1.19(1.07-1.31)	25.6	0.261	⊕⊕⊕⊙Moderate

(2) *Results of A Single-Factor Meta-Analysis of All-Cause Mortality*

**Fig 3:**
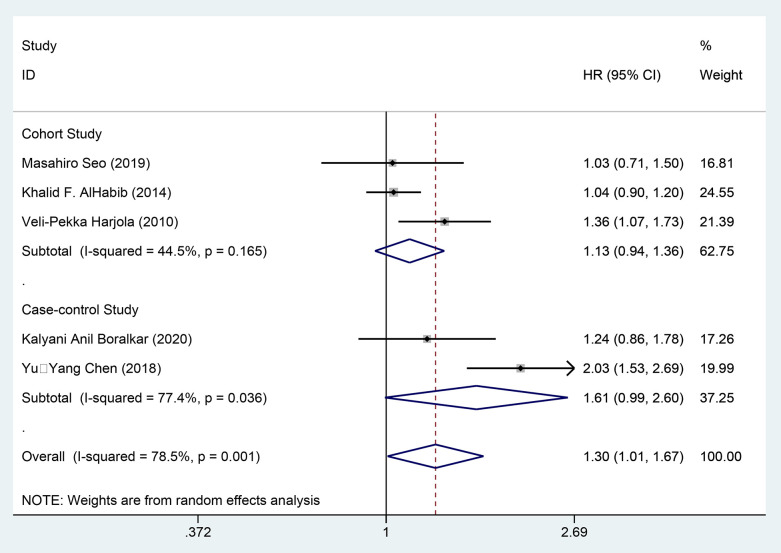
Forest map of univariate meta-analysis of all-cause mortality.

Through univariate analysis, three cohort studies [21,22,25] and two case-control studies [39,44] examined the impact of DM on all-cause mortality among patients with AHF, involving 3,140 patients in the exposure group and 3,781 in the control group. The effect sizes were combined using a random-effects model (*I²* = 78.5%, *P* = 0.001), and a subgroup analysis was conducted based on study type. The findings indicated that in case-control studies [HR = 1.61, 95%CI (0.99, 2.60), GRADE = Low], DM significantly increased the risk of all-cause mortality in patients with AHF. Conversely, in cohort studies [HR = 1.13, 95%CI (0.94, 1.36), GRADE = Moderate], DM did not show a clear connection to all-cause mortality in patients with AHF, as demonstrated in Fig 3 and Table 3.

(3) *Results of Meta-Analysis on Cardiovascular Mortality*

**Fig 4:**
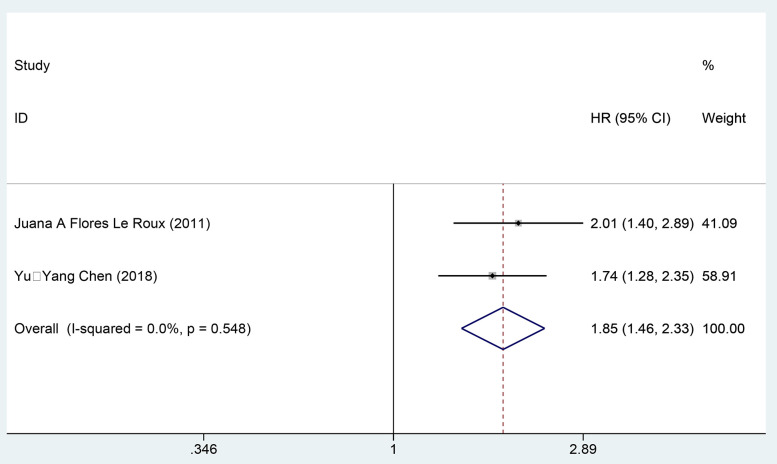
Forest map of meta-analysis of cardiovascular mortality.

Two cohort studies [34,39] employing multivariate analysis examined the impact of DM on cardiovascular mortality among patients with AHF, including 322 individuals in the exposure group and 535 in the control group. Data synthesis was performed using a fixed-effects model (*I²* = 0.0%, *P* = 0.548), revealing that DM significantly heightened the risk of all-cause mortality in AHF patients compared to those without DM [HR = 1.85, 95% CI (1.46, 2.33), GRADE = Moderate], as presented in Fig 4 and Table 3.

(4) *Results of Meta-Analysis on In-Hospital Mortality*

**Fig 5:**
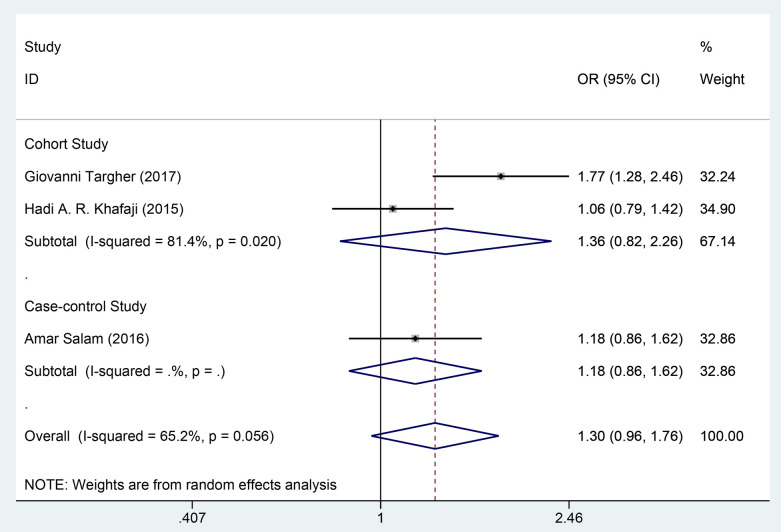
Forest map of multivariate meta-analysis of in-hospital mortality.

Through multivariate analysis, two cohort studies [20,31] and one case-control study [29] investigated the impact of DM on hospital mortality in patients with AHF, involving 8,406 patients in the exposure group and 8,480 in the control group. The data were combined using a random-effects model (*I²* = 65.2%, *P* = 0.056). The meta-analysis results indicated that in both the cohort studies [HR = 1.36, 95%CI (0.82, 2.26), GRADE = Low] and the case-control study [HR = 1.18, 95%CI (0.86, 1.62), GRADE = Low], DM was not significantly related to the risk of in-hospital mortality in patients with AHF, as depicted in Fig 5 and Table 3.

#### 3.4.2 Results of meta-analysis on readmission rate.

**Fig 6:**
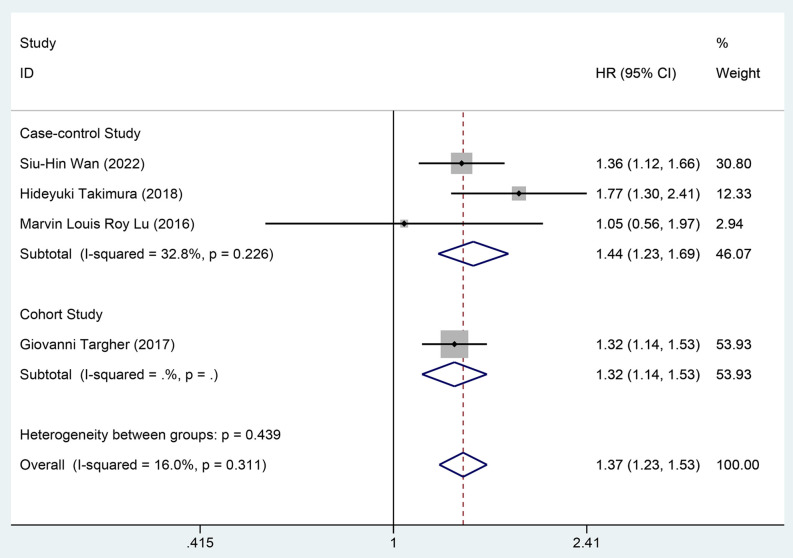
Forest map of multivariate meta-analysis of readmission rate.

Through multivariate analysis, one cohort study [20] and three case-control studies [38,42,43] investigated the impact of DM on the readmission rate in patients with AHF, involving 4,497 patients in the exposure group and 5,143 patients in the control group. The data were combined using a fixed-effects model (*I²* = 16.0%, *P* = 0.311). The meta-analysis indicated that in cohort studies [HR = 1.32, 95%CI (1.14, 1.53), GRADE = Low] and case-control studies [HR = 1.44, 95%CI (1.23, 1.69), GRADE = Moderate], DM significantly elevated the risk of readmission for AHF patients, as demonstrated in Fig 6 and Table 3.

#### 3.4.3 Results of meta-analysis of the co-endpoint of death or readmission.

**Fig 7:**
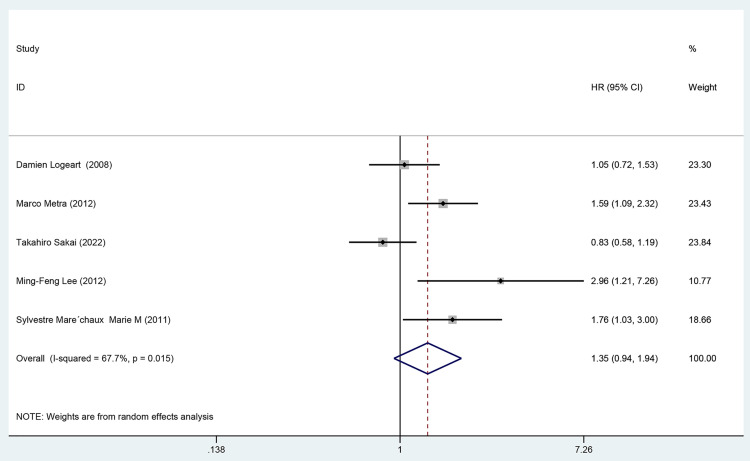
Forest map of univariate meta-analysis of death or readmission.

Five cohort studies [24,28,32,33,35,45] employing univariate analysis assessed the impact of DM on mortality or readmission in patients with AHF, incorporating 381 patients in the exposure group and 841 in the control group. The data were synthesized using a random-effects model (*I²* = 67.7%, *P* = 0.015). These results indicated that, according to cohort studies with univariate analysis [HR = 1.35, 95%CI (0.94, 1.94), GRADE = Low], there was no significant association between DM and the risk of mortality or readmission in patients with AHF, as depicted in Fig 7 and Table 3.

#### 3.4.4 Results of meta-analysis on binary mortality data.

**Fig 8:**
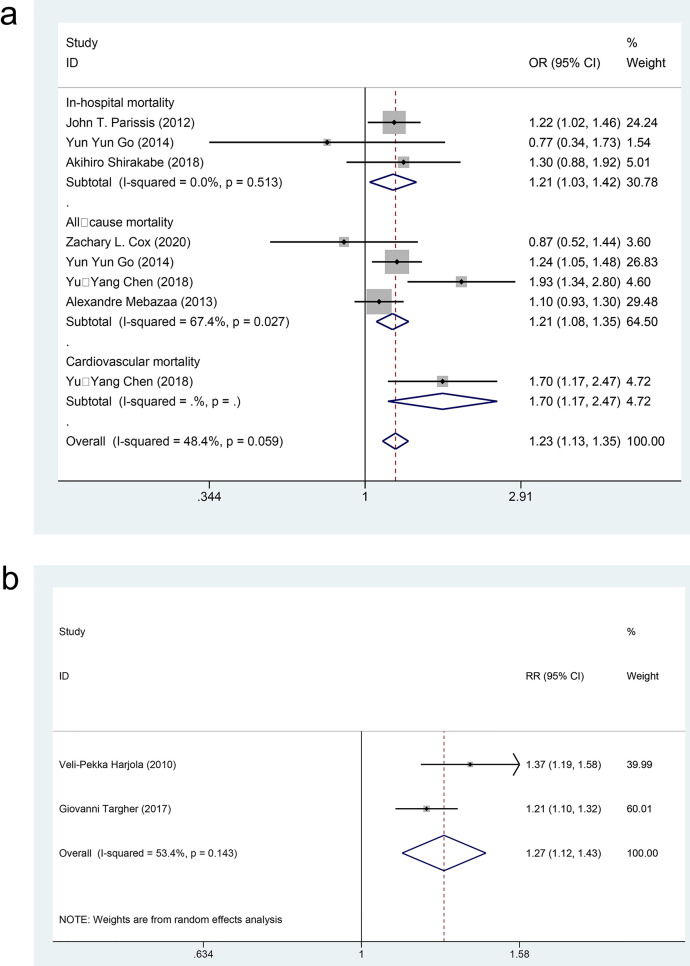
Meta-analysis forest map of mortality as binary data (a) in the cohort studies and (b) in the case-control studies.

Eight case-control studies [36,39,46–49] and two cohort studies [22,50] xamined the impact of DM on mortality among patients with AHF, involving 11,734 patients in the exposure group and 14,254 patients in the control group. The effect sizes were combined using both a fixed-effects model (*I²* = 48.4%, *P* = 0.059) and a random-effects model (*I²* = 53.4%, *P* = 0.143). The findings indicated that in the case-control studies (Fig 8a and Table 3), DM significantly heightened the risk of in-hospital mortality [OR = 1.21, 95%CI (1.03, 1.42), GRADE = Moderate], all-cause mortality [OR = 1.21, 95%CI (1.08, 1.35), GRADE = Low], and cardiovascular mortality [OR = 1.70, 95%CI (1.17, 2.47), GRADE = Low] among AHF patients.

In the cohort studies (Fig 8b and Table 3), DM increased the risk of all-cause mortality in patients with AHF [RR = 1.27, 95%CI (1.12, 1.43), GRADE = Moderate].

#### 3.4.5 Results of meta-analysis on binary readmission rate data.

**Fig 9:**
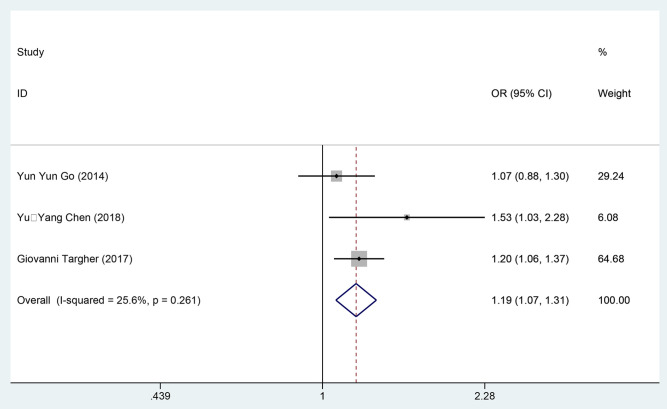
Meta-analysis forest map of readmission rate as binary data.

Three studies [20,37,39] examined the impact of DM on the readmission rates of patients with AHF, involving 4,703 patients in the exposure group and 4,864 patients in the control group. The data were combined using a fixed-effects model (*I²* = 25.6%, *P* = 0.261), and the findings indicated that DM was significantly associated with an increased risk of readmission in AHF patients [OR=1.19, 95%CI (1.07, 1.31), GRADE = Moderate], as presented in Fig 9 and Table 3.

### 3.5 Publication bias and sensitivity analysis

The bias of the included indicators was analyzed as published. A funnel chart intuitively presented the published bias, while Egger’s test assessed the significance of the funnel plots, considering *P* > 0.05 as indicative of no published bias. No publication bias was detected among all the included indicators. Sensitivity analysis also revealed no sensitivity issues within the included indicators. The results of Egger’s test regarding publication bias are displayed in Table 4.

**Table 4:** Egger’s test results for publication bias.

Data Type	Outcome	Std_Eff	Coef.	Std. Err.	t	p
Univariate analysis	All cause death	slope	−0.09	0.32	−0.28	0.80
		bias	2.56	2.69	0.95	0.41
	Death or rehospitalization	slope	−.6776759	0.5085093	−1.33	0.275
		bias	4.146951	2.284547	1.82	0.167
Multivariate analysis	All cause death of cohort studies	slope	0.0556688	0.1007269	0.55	0.600
		bias	1.615217	1.318676	1.22	0.267
	All cause death of case-control studies	slope	0.2491392	0.1612499	1.55	0.183
		bias	0.708184	1.301197	0.54	0.610
Dichotomous analysis	Dichotomy of death of case-control studies	slope	1.141863	0.2036176	5.61	0.001
		bias	0.938023	1.58804	0.59	0.576

## 4 Discussion

Our findings emphasize the significant impact of DM on all-cause mortality, cardiovascular mortality, and readmission rates, while uncovering a complex relationship with in-hospital mortality and composite endpoints. These results not only confirm the pathophysiological interaction between DM and AHF but also highlight important gaps in current management strategies, calling for a paradigm shift towards integrated cardiometabolic care.

Based on the aforementioned core conclusions, a thorough investigation into the contradictory results across different analytical dimensions is necessary, focusing particularly on the heterogeneity observed in all-cause mortality and in-hospital mortality. These findings involve complex methodological challenges and variations in the underlying pathophysiological mechanisms. The following presents a systematic analysis of the potential causes for these two types of contradictory results, considering the characteristics of the research design and the clinical context. Concerning the reasons for the discrepancies in all-cause mortality analysis, it may be attributed to potential associations between diabetes and other confounding variables. The actual impact of diabetes might have been obscured by these confounders in univariate analysis, only to be revealed as having an independent effect on the outcome event after multivariate analysis eliminated the influence of other confounders, thus uncovering the true effect of diabetes on the dependent variable. Regarding the heterogeneity in the analysis of in-hospital mortality, the contradictions predominantly arise from multiple differences in study design and methodology. Firstly, variations in study quality can significantly affect results. For instance, lower-quality studies with inadequate adjustment for confounding variables or insufficient outcome assessment might underestimate the true relationship between diabetes and in-hospital mortality. In our meta-analysis, only one of the six studies reporting in-hospital mortality was rated as high quality, while the others had a moderate risk of bias, potentially leading to inconsistent effect estimates. Secondly, follow-up duration varied significantly across studies (ranging from hospitalization periods to 1-year follow-up). Studies with shorter follow-ups may fail to capture early post-discharge deaths misclassified as non-hospital mortality, whereas longer follow-up studies could obscure the acute metabolic effects of hyperglycemia during hospitalization. Thirdly, selection bias is a major concern. Studies enrolling AHF patients from tertiary centers might include more severe cases with higher baseline mortality, obscuring diabetes-specific risks. Conversely, population-based studies might lack the detail necessary to capture diabetes severity. Some studies only include critically ill patients with AHF, which could obscure the role of diabetes in the general AHF population. Slight differences in any of these aspects can significantly impact the overall results, leading to considerable bias in the study findings. Other researchers have also examined the connection between diabetes and negative prognosis in AHF.

A 2017 meta-analysis regarding diabetes’s prognostic impact on long-term survival outcomes in heart failure patients demonstrated that diabetes adversely affects the long-term survival of AHF patients, significantly raising the risk of all-cause mortality [17]. Nevertheless, their primary focus is on the correlation between diabetes and the negative outcomes of CHF, which include all-cause mortality risk, cardiovascular mortality risk, and the combined endpoint of readmission. The study evidence for the negative prognosis of AHF is very scarce. Among patients with AHF and CHF, there is a considerable variance in the rate of adverse prognosis occurrence [2]. In our study, it was found that the all-cause mortality risk results remained consistent with their conclusions, provided that additional evidence was supplemented. It also highlighted the risks of cardiovascular mortality, in-hospital mortality, readmission, and the composite endpoint of mortality or readmission, further illustrating that diabetes increases the risk of adverse prognosis in AHF.

The association between diabetes and adverse prognosis in AHF patients may be attributed to multiple potential mechanisms. Diabetes exacerbates the onset and progression of AHF through various interconnected pathophysiological mechanisms involving the synergistic effects of hyperglycemia, insulin resistance, and metabolic disturbances on cardiomyocytes, inflammatory pathways, and renal function [7,8,51]. In myocardial fibrosis, chronic hyperglycemia promotes myocardial fibroblast proliferation and abnormal collagen deposition through the activation of the advanced glycation end-product-receptor axis and the transforming growth factor-β/Smad signaling pathway, leading to increased myocardial stiffness and diastolic dysfunction [52,53]. This process is especially critical in the acute decompensated phase as the fibrotic myocardium is unable to effectively regulate changes in volume load, directly triggering pulmonary congestion and a sudden drop in cardiac output, forming the pathological basis of AHF [54]. Concurrently, diabetes-induced mitochondrial dysfunction reduces ATP synthesis in cardiomyocytes, further weakening the heart’s contractile reserve, and the cardiac compensatory mechanism rapidly collapses when exposed to acute triggers like infection and ischemia [55,56]. At the level of inflammatory mechanisms, the chronic low-grade inflammatory state unique to diabetes continuously activates monocytes and macrophages through the NF-κB pathway, releasing pro-inflammatory factors such as TNF-α and IL-6, which not only directly damage the integrity of cardiomyocyte membranes but also trigger oxidative stress via the up-regulation of inducible nitric oxide synthase, leading to dysfunction of sarcoplasmic reticulum calcium-ion regulatory proteins. Ion regulatory proteins function abnormally, exacerbating the reduced amplitude of systolic calcium transients and delayed diastolic calcium clearance [57]. This inflammation-oxidative stress cycle is further amplified in the acute phase of AHF; acute hyperglycemia triggers rapid blood volume changes through osmotic diuresis, activating the renin-angiotensin-aldosterone system and the sympathetic nervous system, increasing inflammatory mediators’ release by 3–5 times, accelerating myocardial apoptosis and microcirculatory disorders, creating a “waterfall effect” of deteriorating cardiac function [44]. Renal dysfunction, a common diabetic complication, contributes to AHF’s pathologic process through a dual mechanism. On one hand, the decrease in glomerular filtration rate and increase in tubular sodium reabsorption due to diabetic nephropathy lead to fluid retention and elevated cardiac preload, directly inducing AHF [28]; on the other hand, the kidneys’ impaired ability to excrete neurohormones leads to abnormally high levels of vasopressin and endothelin-1, aggravating cardiac afterload and promoting myocardial hypertrophy [58,59]. It is noteworthy that diabetic patients have heightened sensitivity to renal hypoxia, and a sharp decline in renal blood flow during an AHF episode can cause acute kidney injury, forming a vicious circle of “cardiorenal syndrome,” decreasing diuretic responsiveness, and further exacerbating fluid overload [60–62]. These three pathways interact at the molecular level; AGEs activate local inflammatory responses in the kidney, and inflammatory factors enhance myocardial fibrosis by up-regulating angiotensin II receptors, constituting a cascading metabolic-structural-functional network of damage unique to diabetes-associated AHF, ultimately leading to significantly higher readmission rates and cardiovascular mortality [63,64].

Our meta-analysis offers several significant advantages. Firstly, it is a systematic review based on a substantial sample size of 326,928, aimed at exploring whether diabetes impacts the survival outcomes of patients with AHF. The high number of total deaths provided sufficient statistical power to quantitatively assess the relationship between diabetes and long-term survival outcomes. Moreover, through the multivariate analysis method, the effects of numerous factors can be examined simultaneously, effectively controlling the influence of confounding factors. Consequently, most researchers in previous studies have utilized the multivariate analysis method when conducting medical statistics. While multivariate analysis holds importance, univariate analysis should not be overlooked. In this regard, our data analysis emphasizes examining multiple factors according to different types of literature. Concurrently, we also presented certain univariate analysis results to enhance the stability and reliability of the findings. Lastly, compared to previous relevant meta-analyses, our study includes more primary studies and discusses a wider range of adverse prognoses.

Our meta-analysis faced some limitations inherent to the nature of the included studies. Firstly, while the data on all-cause mortality were the most comprehensive, information on cardiovascular mortality, in-hospital mortality, and readmission rates was limited. This may be due to some eligible studies having relatively short follow-up periods, with a minimum of just 3 months. Further, most eligible studies did not provide complete data on the duration of diabetes microangiopathy complications, HbA1c levels, use of different types of antidiabetic drugs at baseline, or HbA1c levels during follow-up. Secondly, the diagnosis of diabetes was not always consistent across the included studies. Although the vast majority of cases involved type 2 diabetes, inaccuracies may exist in the estimation of diabetes prevalence and identification of diabetes subtypes. Most heart failure studies primarily enrolled patients with clinically diagnosed diabetes or those using antidiabetic drugs, potentially excluding up to 20% of diabetes patients adhering to dietary recommendations and undiagnosed diabetes patients. Thirdly, the original study lacks explicit data on whether SGLT2i and other antidiabetic drugs reduce the risk of adverse prognosis in patients with AHF who also have diabetes. Consequently, our systematic review did not offer a further in-depth discussion on this matter. Fourth, because the original studies did not extensively address the severity of heart failure, our study was unable to conduct subgroup analyses based on the severity of HF. Fifth, the number of included studies was extremely limited, which prevented the use of Meta-regression to assess the effects of covariates on outcomes.

### 4.1 Prospects

Considering the recent advancements in drug therapy for diabetes [65,66] and the 2021 ESC Heart Failure Guidelines recommendation [2], early identification of certain significant diabetes-related variables, which can provide more precise information on diabetes incidence, may hold clinical importance. Increased focus should be on diagnosing abnormal blood sugar in heart failure patients, as this simple and cost-effective intervention can identify high-risk individuals who could benefit from more active treatment. Consequently, future large-scale randomized controlled trials are necessary to evaluate the impact of intensive blood glucose control on mortality and hospitalization risk in AHF patients.

## 5 Conclusions

This study indicates that exposure to diabetes elevates the risk of adverse outcomes in AHF patients, particularly impacting their survival and hospitalization risk negatively. Consequently, in clinical practice, effective diabetes management and AHF treatment strategies should be developed for these patients to reduce the malignant cycle caused by diabetes and the adverse outcomes of AHF. Furthermore, education and follow-up for diabetic patients should be intensified to quickly identify and address potential complications, thereby enhancing their quality of life and prognosis.

## Supporting information

S1 File PRISMA checklist. (DOCX)

S1 Table Search strategy. (DOCX)

S2 Table Data of each outcome indicator used for meta-analysis. (XLSX)

S3 Table Studies excluded after reading the full text and reasons. (XLSX)
