# Supplementary material for: GV effects of diabetes mellitus on clinical outcomes of patients with acute heart failure: A systematic review and meta-analysis
Source: PLoS One. 2025 Dec 10;20(12):e0338653. doi: 10.1371/journal.pone.0338653 (PMC12694800; doi:10.1371/journal.pone.0338653)
Supplement: S1 Table — (DOCX) [file pone.0338653.s001.docx]

# **Supplementary Table S1. Search strategy**

**1.Pubmed**

| Search number | Query |
| --- | --- |
| #1 | "Heart Failure"[Mesh] |
| #2 | (((((((((((((((((((((((((((Heart Failure[Title/Abstract]) OR (Cardiac Failure[Title/Abstract])) OR (Congestive Heart Failure[Title/Abstract])) OR (Heart Decompensation[Title/Abstract])) OR (Left Sided Heart Failure[Title/Abstract])) OR (Left-Sided Heart Failure[Title/Abstract])) OR (Myocardial Failure[Title/Abstract])) OR (Right Sided Heart Failure[Title/Abstract])) OR (Right-Sided Heart Failure[Title/Abstract])) OR (cardiac backward failure[Title/Abstract])) OR (cardiac decompensation[Title/Abstract])) OR (cardiac failure[Title/Abstract])) OR (cardiac incompetence[Title/Abstract])) OR (cardiac insufficiency[Title/Abstract])) OR (cardiac stand still[Title/Abstract])) OR (cardial decompensation[Title/Abstract])) OR (cardial insufficiency[Title/Abstract])) OR (chronic heart failure[Title/Abstract])) OR (chronic heart insufficiency[Title/Abstract])) OR (decompensatio cordis[Title/Abstract])) OR (heart backward failure[Title/Abstract])) OR (heart decompensation[Title/Abstract])) OR (heart incompetence[Title/Abstract])) OR (heart insufficiency[Title/Abstract])) OR (insufficientia cardis[Title/Abstract])) OR (myocardial failure[Title/Abstract])) OR (myocardial insufficiency[Title/Abstract])) OR ("Heart Failure"[Mesh]) |
| #3 | ((((((((((((((((((((((((((((((((((((((((((((((((((Diabetes Mellitus[MeSH Terms]) OR (Diabetes Mellitus[Title/Abstract])) OR (diabetes[Title/Abstract])) OR (diabetic[Title/Abstract])) OR (Diabetes Mellitus, Type 1[MeSH Terms])) OR (Diabetes Mellitus, Type 1[Title/Abstract])) OR (Autoimmune Diabetes[Title/Abstract])) OR (Brittle Diabetes Mellitus[Title/Abstract])) OR (IDDM[Title/Abstract])) OR (Insulin Dependent Diabetes Mellitus 1[Title/Abstract])) OR (Insulin-Dependent Diabetes Mellitus[Title/Abstract])) OR (Juvenile Onset Diabetes[Title/Abstract])) OR (Ketosis-Prone Diabetes Mellitus[Title/Abstract])) OR (Sudden-Onset Diabetes Mellitus[Title/Abstract])) OR (Type 1 Diabetes[Title/Abstract])) OR (insulin dependent diabetes mellitus[Title/Abstract])) OR (diabetes mellitus type 1[Title/Abstract])) OR (diabetes mellitus type i[Title/Abstract])) OR (diabetes type 1[Title/Abstract])) OR (diabetes type I[Title/Abstract])) OR (dm 1[Title/Abstract])) OR (early onset diabetes mellitus[Title/Abstract])) OR (insulin dependent diabetes[Title/Abstract])) OR (juvenile diabetes[Title/Abstract])) OR (ketoacidotic diabetes[Title/Abstract])) OR (labile diabetes mellitus[Title/Abstract])) OR (mckusick 22210[Title/Abstract])) OR (T1DM[Title/Abstract])) OR (type I diabetes[Title/Abstract])) OR (Diabetes Mellitus, Type 2[MeSH Terms])) OR (Diabetes Mellitus, Type 2[Title/Abstract])) OR (Adult-Onset Diabetes Mellitus[Title/Abstract])) OR (Ketosis-Resistant Diabetes Mellitus[Title/Abstract])) OR (Maturity Onset Diabetes[Title/Abstract])) OR (MODY[Title/Abstract])) OR (NIDDM[Title/Abstract])) OR (Noninsulin Dependent Diabetes Mellitus[Title/Abstract])) OR (Slow-Onset Diabetes Mellitus[Title/Abstract])) OR (Stable Diabetes Mellitus[Title/Abstract])) OR (Type 2 Diabetes[Title/Abstract])) OR (non insulin dependent diabetes mellitus[Title/Abstract])) OR (adult onset diabetes[Title/Abstract])) OR (diabetes mellitus type ii[Title/Abstract])) OR (diabetes type 2[Title/Abstract])) OR (diabetes type II[Title/Abstract])) OR (dm 2[Title/Abstract])) OR (insulin independent diabetes[Title/Abstract])) OR (ketosis resistant diabetes mellitus[Title/Abstract])) OR (non insulin dependent diabetes[Title/Abstract])) OR (T2DM[Title/Abstract])) OR (type II diabetes[Title/Abstract]) |
| #4 | (((((((((((((((((((((((((((((((((((((((((((((((((((Diabetes Mellitus[MeSH Terms]) OR (Diabetes Mellitus[Title/Abstract])) OR (diabetes[Title/Abstract])) OR (diabetic[Title/Abstract])) OR (Diabetes Mellitus, Type 1[MeSH Terms])) OR (Diabetes Mellitus, Type 1[Title/Abstract])) OR (Autoimmune Diabetes[Title/Abstract])) OR (Brittle Diabetes Mellitus[Title/Abstract])) OR (IDDM[Title/Abstract])) OR (Insulin Dependent Diabetes Mellitus 1[Title/Abstract])) OR (Insulin-Dependent Diabetes Mellitus[Title/Abstract])) OR (Juvenile Onset Diabetes[Title/Abstract])) OR (Ketosis-Prone Diabetes Mellitus[Title/Abstract])) OR (Sudden-Onset Diabetes Mellitus[Title/Abstract])) OR (Type 1 Diabetes[Title/Abstract])) OR (insulin dependent diabetes mellitus[Title/Abstract])) OR (diabetes mellitus type 1[Title/Abstract])) OR (diabetes mellitus type i[Title/Abstract])) OR (diabetes type 1[Title/Abstract])) OR (diabetes type I[Title/Abstract])) OR (dm 1[Title/Abstract])) OR (early onset diabetes mellitus[Title/Abstract])) OR (insulin dependent diabetes[Title/Abstract])) OR (juvenile diabetes[Title/Abstract])) OR (ketoacidotic diabetes[Title/Abstract])) OR (labile diabetes mellitus[Title/Abstract])) OR (mckusick 22210[Title/Abstract])) OR (T1DM[Title/Abstract])) OR (type I diabetes[Title/Abstract])) OR (Diabetes Mellitus, Type 2[MeSH Terms])) OR (Diabetes Mellitus, Type 2[Title/Abstract])) OR (Adult-Onset Diabetes Mellitus[Title/Abstract])) OR (Ketosis-Resistant Diabetes Mellitus[Title/Abstract])) OR (Maturity Onset Diabetes[Title/Abstract])) OR (MODY[Title/Abstract])) OR (NIDDM[Title/Abstract])) OR (Noninsulin Dependent Diabetes Mellitus[Title/Abstract])) OR (Slow-Onset Diabetes Mellitus[Title/Abstract])) OR (Stable Diabetes Mellitus[Title/Abstract])) OR (Type 2 Diabetes[Title/Abstract])) OR (non insulin dependent diabetes mellitus[Title/Abstract])) OR (adult onset diabetes[Title/Abstract])) OR (diabetes mellitus type ii[Title/Abstract])) OR (diabetes type 2[Title/Abstract])) OR (diabetes type II[Title/Abstract])) OR (dm 2[Title/Abstract])) OR (insulin independent diabetes[Title/Abstract])) OR (ketosis resistant diabetes mellitus[Title/Abstract])) OR (non insulin dependent diabetes[Title/Abstract])) OR (T2DM[Title/Abstract])) OR (type II diabetes[Title/Abstract])) AND ((((((((((((((((((((((((((((Heart Failure[Title/Abstract]) OR (Cardiac Failure[Title/Abstract])) OR (Congestive Heart Failure[Title/Abstract])) OR (Heart Decompensation[Title/Abstract])) OR (Left Sided Heart Failure[Title/Abstract])) OR (Left-Sided Heart Failure[Title/Abstract])) OR (Myocardial Failure[Title/Abstract])) OR (Right Sided Heart Failure[Title/Abstract])) OR (Right-Sided Heart Failure[Title/Abstract])) OR (cardiac backward failure[Title/Abstract])) OR (cardiac decompensation[Title/Abstract])) OR (cardiac failure[Title/Abstract])) OR (cardiac incompetence[Title/Abstract])) OR (cardiac insufficiency[Title/Abstract])) OR (cardiac stand still[Title/Abstract])) OR (cardial decompensation[Title/Abstract])) OR (cardial insufficiency[Title/Abstract])) OR (chronic heart failure[Title/Abstract])) OR (chronic heart insufficiency[Title/Abstract])) OR (decompensatio cordis[Title/Abstract])) OR (heart backward failure[Title/Abstract])) OR (heart decompensation[Title/Abstract])) OR (heart incompetence[Title/Abstract])) OR (heart insufficiency[Title/Abstract])) OR (insufficientia cardis[Title/Abstract])) OR (myocardial failure[Title/Abstract])) OR (myocardial insufficiency[Title/Abstract])) OR ("Heart Failure"[Mesh])) |
| #5 | (((((((((((((Treatment Outcome[MeSH Terms]) OR (Treatment Outcome[Title/Abstract])) OR (Clinical Effectiveness[Title/Abstract])) OR (Clinical Efficacy[Title/Abstract])) OR (Outcome, Treatment[Title/Abstract])) OR (Patient Relevant Outcome[Title/Abstract])) OR (Rehabilitation Outcome[Title/Abstract])) OR (Treatment Effectiveness[Title/Abstract])) OR (Treatment Efficacy[Title/Abstract])) OR (clinical outcome[Title/Abstract])) OR (clinical patient outcome[Title/Abstract])) OR (clinical therapeutic outcome[Title/Abstract])) OR (clinical therapy outcome[Title/Abstract])) OR (clinical treatment outcome[Title/Abstract]) |
| #6 | ((((((((((((((Treatment Outcome[MeSH Terms]) OR (Treatment Outcome[Title/Abstract])) OR (Clinical Effectiveness[Title/Abstract])) OR (Clinical Efficacy[Title/Abstract])) OR (Outcome, Treatment[Title/Abstract])) OR (Patient Relevant Outcome[Title/Abstract])) OR (Rehabilitation Outcome[Title/Abstract])) OR (Treatment Effectiveness[Title/Abstract])) OR (Treatment Efficacy[Title/Abstract])) OR (clinical outcome[Title/Abstract])) OR (clinical patient outcome[Title/Abstract])) OR (clinical therapeutic outcome[Title/Abstract])) OR (clinical therapy outcome[Title/Abstract])) OR (clinical treatment outcome[Title/Abstract])) AND ((((((((((((((((((((((((((((((((((((((((((((((((((((Diabetes Mellitus[MeSH Terms]) OR (Diabetes Mellitus[Title/Abstract])) OR (diabetes[Title/Abstract])) OR (diabetic[Title/Abstract])) OR (Diabetes Mellitus, Type 1[MeSH Terms])) OR (Diabetes Mellitus, Type 1[Title/Abstract])) OR (Autoimmune Diabetes[Title/Abstract])) OR (Brittle Diabetes Mellitus[Title/Abstract])) OR (IDDM[Title/Abstract])) OR (Insulin Dependent Diabetes Mellitus 1[Title/Abstract])) OR (Insulin-Dependent Diabetes Mellitus[Title/Abstract])) OR (Juvenile Onset Diabetes[Title/Abstract])) OR (Ketosis-Prone Diabetes Mellitus[Title/Abstract])) OR (Sudden-Onset Diabetes Mellitus[Title/Abstract])) OR (Type 1 Diabetes[Title/Abstract])) OR (insulin dependent diabetes mellitus[Title/Abstract])) OR (diabetes mellitus type 1[Title/Abstract])) OR (diabetes mellitus type i[Title/Abstract])) OR (diabetes type 1[Title/Abstract])) OR (diabetes type I[Title/Abstract])) OR (dm 1[Title/Abstract])) OR (early onset diabetes mellitus[Title/Abstract])) OR (insulin dependent diabetes[Title/Abstract])) OR (juvenile diabetes[Title/Abstract])) OR (ketoacidotic diabetes[Title/Abstract])) OR (labile diabetes mellitus[Title/Abstract])) OR (mckusick 22210[Title/Abstract])) OR (T1DM[Title/Abstract])) OR (type I diabetes[Title/Abstract])) OR (Diabetes Mellitus, Type 2[MeSH Terms])) OR (Diabetes Mellitus, Type 2[Title/Abstract])) OR (Adult-Onset Diabetes Mellitus[Title/Abstract])) OR (Ketosis-Resistant Diabetes Mellitus[Title/Abstract])) OR (Maturity Onset Diabetes[Title/Abstract])) OR (MODY[Title/Abstract])) OR (NIDDM[Title/Abstract])) OR (Noninsulin Dependent Diabetes Mellitus[Title/Abstract])) OR (Slow-Onset Diabetes Mellitus[Title/Abstract])) OR (Stable Diabetes Mellitus[Title/Abstract])) OR (Type 2 Diabetes[Title/Abstract])) OR (non insulin dependent diabetes mellitus[Title/Abstract])) OR (adult onset diabetes[Title/Abstract])) OR (diabetes mellitus type ii[Title/Abstract])) OR (diabetes type 2[Title/Abstract])) OR (diabetes type II[Title/Abstract])) OR (dm 2[Title/Abstract])) OR (insulin independent diabetes[Title/Abstract])) OR (ketosis resistant diabetes mellitus[Title/Abstract])) OR (non insulin dependent diabetes[Title/Abstract])) OR (T2DM[Title/Abstract])) OR (type II diabetes[Title/Abstract])) AND ((((((((((((((((((((((((((((Heart Failure[Title/Abstract]) OR (Cardiac Failure[Title/Abstract])) OR (Congestive Heart Failure[Title/Abstract])) OR (Heart Decompensation[Title/Abstract])) OR (Left Sided Heart Failure[Title/Abstract])) OR (Left-Sided Heart Failure[Title/Abstract])) OR (Myocardial Failure[Title/Abstract])) OR (Right Sided Heart Failure[Title/Abstract])) OR (Right-Sided Heart Failure[Title/Abstract])) OR (cardiac backward failure[Title/Abstract])) OR (cardiac decompensation[Title/Abstract])) OR (cardiac failure[Title/Abstract])) OR (cardiac incompetence[Title/Abstract])) OR (cardiac insufficiency[Title/Abstract])) OR (cardiac stand still[Title/Abstract])) OR (cardial decompensation[Title/Abstract])) OR (cardial insufficiency[Title/Abstract])) OR (chronic heart failure[Title/Abstract])) OR (chronic heart insufficiency[Title/Abstract])) OR (decompensatio cordis[Title/Abstract])) OR (heart backward failure[Title/Abstract])) OR (heart decompensation[Title/Abstract])) OR (heart incompetence[Title/Abstract])) OR (heart insufficiency[Title/Abstract])) OR (insufficientia cardis[Title/Abstract])) OR (myocardial failure[Title/Abstract])) OR (myocardial insufficiency[Title/Abstract])) OR ("Heart Failure"[Mesh]))) |

**Cochrane**

| Search number | Query |
| --- | --- |
| #1 | MeSH descriptor: [Diabetes Mellitus] explode all trees |
| #2 | (Diabetes Mellitus):ti,ab,kw OR (diabetes):ti,ab,kw OR (diabetic):ti,ab,kw |
| #3 | MeSH descriptor: [Diabetes Mellitus, Type 1] explode all trees |
| #4 | (Diabetes Mellitus, Type 1):ti,ab,kw OR (Autoimmune Diabetes):ti,ab,kw OR (Brittle Diabetes Mellitus):ti,ab,kw OR (IDDM):ti,ab,kw OR (Insulin Dependent Diabetes Mellitus 1):ti,ab,kw |
| #5 | (Insulin-Dependent Diabetes Mellitus):ti,ab,kw OR (Juvenile Onset Diabetes):ti,ab,kw OR (Ketosis-Prone Diabetes Mellitus):ti,ab,kw OR (Sudden-Onset Diabetes Mellitus):ti,ab,kw OR (Type 1 Diabetes):ti,ab,kw |
| #6 | (insulin dependent diabetes mellitus):ti,ab,kw OR (diabetes mellitus type 1):ti,ab,kw OR (diabetes mellitus type i):ti,ab,kw OR (diabetes type 1):ti,ab,kw OR (diabetes type I):ti,ab,kw |
| #7 | (dm 1):ti,ab,kw OR (early onset diabetes mellitus):ti,ab,kw OR (insulin dependent diabetes):ti,ab,kw OR (juvenile diabetes):ti,ab,kw OR (ketoacidotic diabetes):ti,ab,kw |
| #8 | (labile diabetes mellitus):ti,ab,kw OR (mckusick 22210):ti,ab,kw OR (T1DM):ti,ab,kw OR (type I diabetes):ti,ab,kw |
| #9 | MeSH descriptor: [Diabetes Mellitus, Type 2] explode all trees |
| #10 | (Diabetes Mellitus, Type 2):ti,ab,kw OR (Adult-Onset Diabetes Mellitus):ti,ab,kw OR (Ketosis-Resistant Diabetes Mellitus):ti,ab,kw OR (Maturity Onset Diabetes):ti,ab,kw OR (MODY):ti,ab,kw |
| #11 | (NIDDM):ti,ab,kw OR (Noninsulin Dependent Diabetes Mellitus):ti,ab,kw OR (Slow-Onset Diabetes Mellitus):ti,ab,kw OR (Stable Diabetes Mellitus):ti,ab,kw OR (Type 2 Diabetes):ti,ab,kw |
| #12 | #1 or #2 or #3 or #4 or #5 or #6 or #7 or #8 or #9 or #10 or #11 |
| #13 | MeSH descriptor: [Heart Failure] explode all trees |
| #14 | (Heart Failure):ti,ab,kw OR (Cardiac Failure):ti,ab,kw OR (Congestive Heart Failure):ti,ab,kw OR (Heart Decompensation):ti,ab,kw OR (Left Sided Heart Failure):ti,ab,kw |
| #15 | (Myocardial Failure):ti,ab,kw OR (Right Sided Heart Failure):ti,ab,kw OR (cardiac backward failure):ti,ab,kw OR (cardiac decompensation):ti,ab,kw OR (cardiac incompetence):ti,ab,kw |
| #16 | (cardiac insufficiency):ti,ab,kw OR (cardiac stand still):ti,ab,kw OR (cardial decompensation):ti,ab,kw OR (cardial insufficiency):ti,ab,kw OR (chronic heart failure):ti,ab,kw |
| #17 | (chronic heart insufficiency):ti,ab,kw OR (decompensatio cordis):ti,ab,kw OR (heart backward failure):ti,ab,kw OR (heart decompensation):ti,ab,kw OR (heart incompetence):ti,ab,kw |
| #18 | (heart insufficiency):ti,ab,kw OR (insufficientia cardis):ti,ab,kw OR (myocardial failure):ti,ab,kw OR (myocardial insufficiency):ti,ab,kw |
| #19 | #13 or #14 or #15 or #16 or #17 or #18 |
| #20 | MeSH descriptor: [Treatment Outcome] explode all trees |
| #21 | (Treatment Outcome):ti,ab,kw OR (Clinical Effectiveness):ti,ab,kw OR (Clinical Efficacy):ti,ab,kw OR (Outcome, Treatment):ti,ab,kw OR (Patient Relevant Outcome):ti,ab,kw |
| #22 | (Rehabilitation Outcome):ti,ab,kw OR (Treatment Effectiveness):ti,ab,kw OR (Treatment Efficacy):ti,ab,kw OR (clinical outcome):ti,ab,kw OR (clinical patient outcome):ti,ab,kw |
| #23 | (clinical therapeutic outcome):ti,ab,kw OR (clinical therapy outcome):ti,ab,kw OR (clinical treatment outcome):ti,ab,kw |
| #24 | #20 or #21 or #22 or #23 |
| #25 | #12 and #19 and #24 |

**3.Embase**

| Search number | Query |
| --- | --- |
| #1 | 'clinical outcome'/exp OR 'clinical patient  outcome':ti,ab,kw OR 'clinical therapeutic  outcome':ti,ab,kw OR 'clinical therapy  outcome':ti,ab,kw OR 'clinical treatment  outcome':ti,ab,kw OR 'treatment outcome':ti,ab,kw  OR 'clinical effectiveness':ti,ab,kw OR  'clinical efficacy':ti,ab,kw OR 'outcome,  treatment':ti,ab,kw OR 'patient  relevant outcome':ti,ab,kw OR  'rehabilitation outcome':ti,ab,kw OR 'treatment  effectiveness':ti,ab,kw OR 'treatment  efficacy':ti,ab,kw |
| #2 | 'heart failure'/exp OR 'congestive heart  failure':ti,ab,kw OR 'left sided heart  failure':ti,ab,kw OR 'right sided heart  failure':ti,ab,kw OR 'cardiac backward  failure':ti,ab,kw OR 'cardiac  decompensation':ti,ab,kw OR 'cardiac  failure':ti,ab,kw OR 'cardiac  incompetence':ti,ab,kw OR 'cardiac  insufficiency':ti,ab,kw OR 'cardiac stand  still':ti,ab,kw OR 'cardial  decompensation':ti,ab,kw OR 'chronic heart  failure':ti,ab,kw OR 'cardial  insufficiency':ti,ab,kw OR 'chronic heart  insufficiency':ti,ab,kw OR 'decompensatio  cordis':ti,ab,kw OR 'heart backward  failure':ti,ab,kw OR 'heart  decompensation':ti,ab,kw OR 'heart  incompetence':ti,ab,kw OR 'heart  insufficiency':ti,ab,kw OR 'insufficientia  cardis':ti,ab,kw OR 'myocardial failure':ti,ab,kw |
| #3 | 'diabetes mellitus'/exp OR 'insulin dependent  diabetes mellitus'/exp OR 'non insulin dependent  diabetes mellitus'/exp OR diabetes:ti,ab,kw OR  diabetic:ti,ab,kw OR  'autoimmune diabetes':ti,ab,kw OR  'brittle diabetes mellitus':ti,ab,kw OR  iddm:ti,ab,kw OR 'insulin dependent diabetes  mellitus 1':ti,ab,kw OR  'insulin-dependent diabetes mellitus':ti,ab,kw OR  'juvenile onset diabetes':ti,ab,kw OR  'ketosis-prone diabetes mellitus':ti,ab,kw OR  'sudden-onset diabetes mellitus':ti,ab,kw OR  'type 1 diabetes':ti,ab,kw OR  'adult-onset diabetes mellitus':ti,ab,kw OR  'ketosis-resistant diabetes mellitus':ti,ab,kw OR  'maturity onset diabetes':ti,ab,kw OR  mody:ti,ab,kw OR niddm:ti,ab,kw OR 'noninsulin  dependent diabetes mellitus':ti,ab,kw OR  'slow-onset diabetes mellitus':ti,ab,kw OR  'stable diabetes mellitus':ti,ab,kw OR 'type  2 diabetes':ti,ab,kw OR 'diabetes mellitus type  1':ti,ab,kw OR 'diabetes mellitus type  i':ti,ab,kw OR 'diabetes type 1':ti,ab,kw OR  'diabetes type i':ti,ab,kw OR 'dm 1':ti,ab,kw OR  'early onset diabetes mellitus':ti,ab,kw OR  'insulin dependent diabetes':ti,ab,kw OR  'juvenile diabetes':ti,ab,kw OR 'ketoacidotic  diabetes':ti,ab,kw OR 'labile diabetes  mellitus':ti,ab,kw OR 'mckusick 22210':ti,ab,kw  OR t1dm:ti,ab,kw OR 'type i diabetes':ti,ab,kw OR  'adult onset diabetes':ti,ab,kw OR 'diabetes  mellitus type ii':ti,ab,kw OR 'diabetes type  2':ti,ab,kw OR 'diabetes type ii':ti,ab,kw OR 'dm  2':ti,ab,kw OR 'insulin independent  diabetes':ti,ab,kw OR 'non insulin dependent  diabetes':ti,ab,kw OR t2dm:ti,ab,kw OR 'type 2  diabetes':ti,ab,kw OR 'type ii diabetes':ti,ab,kw |
| #4 | #1 AND #2 AND #3 |

**4.Web of science**

| Search number | Query |
| --- | --- |
| #1 | Heart Failure (Topic) or Cardiac Failure (Topic) or Congestive Heart Failure (Topic) or Heart Decompensation (Topic) or Left Sided Heart Failure (Topic) or Myocardial Failure (Topic) or Right Sided Heart Failure (Topic) or cardiac backward failure (Topic) or cardiac decompensation (Topic) or cardiac incompetence (Topic) or cardiac insufficiency (Topic) or cardiac stand still (Topic) or cardial decompensation (Topic) or cardial insufficiency (Topic) or chronic heart failure (Topic) or chronic heart insufficiency (Topic) or decompensatio cordis (Topic) or heart backward failure (Topic) or heart decompensation (Topic) or heart incompetence (Topic) or heart insufficiency (Topic) or insufficientia cardis (Topic) or myocardial failure (Topic) or myocardial insufficiency (Topic) |
| #2 | Diabetes Mellitus (Topic) or diabetes (Topic) or diabetic (Topic) or Diabetes Mellitus, Type 1 (Topic) or Autoimmune Diabetes (Topic) or Brittle Diabetes Mellitus (Topic) or IDDM (Topic) or Insulin Dependent Diabetes Mellitus 1 (Topic) or Insulin-Dependent Diabetes Mellitus (Topic) or Juvenile Onset Diabetes (Topic) or Ketosis-Prone Diabetes Mellitus (Topic) or Sudden-Onset Diabetes Mellitus (Topic) or Type 1 Diabetes (Topic) or Diabetes Mellitus, Type 2 (Topic) or Adult-Onset Diabetes Mellitus (Topic) or Ketosis-Resistant Diabetes Mellitus (Topic) or Maturity Onset Diabetes (Topic) or MODY (Topic) or NIDDM (Topic) or Noninsulin Dependent Diabetes Mellitus (Topic) or Slow-Onset Diabetes Mellitus (Topic) or Stable Diabetes Mellitus (Topic) or Type 2 Diabetes (Topic) or insulin dependent diabetes mellitus (Topic) or diabetes mellitus type 1 (Topic) or diabetes mellitus type i (Topic) or diabetes type 1 (Topic) or diabetes type I (Topic) or dm 1 (Topic) or early onset diabetes mellitus (Topic) or insulin dependent diabetes (Topic) or juvenile diabetes (Topic) or ketoacidotic diabetes (Topic) or labile diabetes mellitus (Topic) or mckusick 22210 (Topic) or T1DM (Topic) or type I diabetes (Topic) or non insulin dependent diabetes mellitus (Topic) or adult onset diabetes (Topic) or diabetes mellitus type ii (Topic) or diabetes type 2 (Topic) or diabetes type II (Topic) or dm 2 (Topic) or insulin independent diabetes (Topic) or non insulin dependent diabetes (Topic) or T2DM (Topic) or type 2 diabetes (Topic) or type II diabetes (Topic) |
| #3 | Treatment Outcome (Topic) or Clinical Effectiveness (Topic) or Clinical Efficacy (Topic) or Outcome, Treatment (Topic) or Patient Relevant Outcome (Topic) or Rehabilitation Outcome (Topic) or Treatment Effectiveness (Topic) or Treatment Efficacy (Topic) or clinical outcome (Topic) or clinical patient outcome (Topic) or clinical therapeutic outcome (Topic) or clinical therapy outcome (Topic) or clinical treatment outcome (Topic) |
| #4 | #1 AND #2 AND #3 |
